# The Use of the Situation, Background, Assessment, and Recommendation (SBAR) Form as a Tool for Handoff Communication in the Pediatrics Department in a Sudanese Teaching Hospital

**DOI:** 10.7759/cureus.31998

**Published:** 2022-11-29

**Authors:** Mosab H Adam, Hiba A Ali, Abubaker Koko, Malaz F Ibrahim, Rasha S Omar, Duaa S Mahmoud, Sara Omer Abdelazim Mohammed, Rana A Ahmed, Kirlus R Habib, Duaa Y Ali

**Affiliations:** 1 Orthopedics, University of Khartoum, Khartoum, SDN; 2 Anatomy, Faculty of Medicine, University of Khartoum, Khartoum, SDN; 3 Biostatistics, Faculty of Medicine, University of Khartoum, Khartoum, SDN; 4 Internal Medicine, University of Khartoum, Khartoum, SDN; 5 Pediatrics, Ribat Teaching Hospital, Khartoum, SDN; 6 Psychiatry, University of Khartoum, Khartoum, SDN; 7 Obstetrics and Gynaecology, National University of Sudan, Khartoum, SDN; 8 Pediatrics, AL-Neelain University, Khartoum, SDN; 9 Surgery, National University of Sudan, Khartoum, SDN

**Keywords:** who, safe patient handoff, pediatrics, sbar, handoff communication

## Abstract

Purpose: Patient care transitions between healthcare providers are common in hospitals -- this project aimed to audit the use of Situation, Background, Assessment, and Recommendation (SBAR)-based handoff communication tool in the handoff process.

Materials and Methods: This prospective audit was conducted at Ribat Teaching Hospital, Khartoum, Sudan. All handoff communications of pediatric inpatients who required close monitoring during the study period were included. Two cycles of data collection were conducted, each spanning a duration of two weeks. The data, whether in the first or second cycle, were collected using a checklist document containing the items of the SBAR form. After the end of the first cycle, regular training sessions about the content and importance of the SBAR form were conducted for one week. Additionally, doctors' perception regarding the form was assessed.

Results: Some 48 doctors participated in this study, 29 females and 19 males. In the first cycle, the percentages of filled SBAR form components were as follows: Situation 7%, Background 0.00%, Assessment 0.00%, and Recommendation 0.00%. After conducting training sessions, the second cycle assessment was done and it showed improvement in all form components: Situation 88.8%, Background 83.6%, Assessment 66.3%, and Recommendation 69.5%. Regarding the doctors’ perception assessment, the majority reported the usefulness of the SBAR form in patients' safety, physicians' communication, and accountability.

Conclusion: The SBAR form is a simple and effective tool for improving communication; it helps doctors capture all relevant patient information. Most importantly, the majority of doctors were satisfied with the use of this tool for handoff communication.

## Introduction

In-hospital patient care involves necessary interactions between several healthcare professionals over successive shifts and across different departments [1]. Adequate and effective communication and handoff information between healthcare professionals has been recognized as an important factor in healthcare quality and patient safety. However, with each handoff, loss of information and miscommunication is expected due to a large amount of information, high frequency of exchange, and differences in perception of what is important and worth mentioning in the handoff process. Furthermore, the stress and exhausting nature of the job render healthcare professionals more prone to making mistakes which may lead to delays in management, prolonged hospitalization, and medication errors compromising patient safety [2-3]. Communication errors have been reported to be one of the major factors leading to sentinel events, as cited by the Sentinel Event Data Report published by the Joint Commission in 2015. A sentinel event is the harm or death of a patient which is not caused by a natural disease process [4]. To reduce the magnitude of this problem, many hospitals use standardized handoff communication -- “a process in which patient’s information is communicated from one healthcare giver to another in a consistent manner.” [5-6]

 The World Health Organization (WHO) and National Health Service in the UK recommend using the SBAR format (Situation, Background, Assessment, and Recommendation) to handle patients’ information between different units and healthcare professionals [7]. The practices of handoff communications have been noted to be conducted in a non-structured, sometimes arbitrary manner [5]. This audit aimed to assess the current handoff practices, evaluate doctors’ perception on using this form, and determine the efficacy of training sessions on the use of the SBAR form in improving handoff communication practices by reassessing the handoff practices after the completion of those sessions.

## Materials and methods

Study design

This is a prospective audit that aimed to evaluate and improve the handoff practices among doctors working in the pediatric department at Ribat Teaching Hospital, Khartoum, Sudan. The audit comprised a first cycle assessing the pre-existing handoff practices of clinical cases in the pediatric department. This assessment was followed by educational and legislative sessions targeting doctors practicing in the pediatric ward, regarding ideal patients' handoff practices. Subsequently, the second cycle of assessment was conducted to gauge the impact of the introduced measures on patients' handoff practices. 

Ethical considerations

This study was considered a quality improvement project. Thus, it was reviewed and approved by the quality improvement center and the pediatrics department. Informed verbal consent was obtained from all doctors involved in handoff processes in the pediatric department. In addition, consent from parents or guardians was obtained to access clinical information recorded in patients’ charts.

 Population and sampling

The study population included all doctors' handoffs of pediatric inpatients who were admitted to the pediatric ward and at risk of their condition deteriorating, and those recently relocated from higher levels of care, whose needs could be met in an acute ward (Level 1 critical care) [8]. We excluded handoffs of patients with stable clinical statuses. Doctors performing handoffs included interns, medical officers, and registrars. Handoff processes between different units during a period of one month (two weeks for each cycle) were included in the assessment.

 Study area

There are seven units in the pediatrics department at Ribat Teaching Hospital, Khartoum, Sudan. Each unit consists of one consultant or specialist, registrars, medical officers, and house officers.

SBAR sheet

The Joint Commission describes the divisions of the SBAR written communication tool as, Situation: what is the situation; why are you handling this patient to the physician? Background: what is the background information? Assessment: what is your assessment of the problem? Recommendation: how should the problem be corrected? After discussion and consensus with the department consultants and stakeholders, modifications in the design of the SBAR form were made in order to make it simpler, more organized, and more efficient in transferring information.

 Data collection

The data collection team assessed the pre-existing written communication tool and documented the patient information written by doctors during handoff communication. Data collection of the first cycle lasted for two weeks in December. Results of the first cycle were presented and discussed during the weekly morbidity and mortality meeting held by the department in January. Subsequently, an intervention in the form of regular training sessions focusing on SBAR handoff was conducted for a period of one week following the first cycle assessment. Data collection of the second cycle (following the intervention) was conducted in February (one month after the completion of the training sessions), which lasted for two weeks. This data -- whether in the first or second cycle -- was collected using a checklist document containing the same items of the SBAR form, as follows: if the doctor documented the specified item, the data collector marked this item as 1; if he/she did not document it, then it was marked as 0; if the item was not applicable, it was marked as 9. Doctors' satisfaction regarding the use of the SBAR form was assessed using an online google form. The collected data was then exported to an excel sheet to be analyzed.

Training sessions

One week after the completion of the first cycle and before commencing the second cycle, daily training sessions and focused group discussions were conducted in all departments’ units. These sessions were organized by the audit team in liaison with the pediatric department. The sessions were conducted daily over a period of one-week lasting one hour each, to ensure the full coverage of all doctors practicing in the pediatric department. The sessions focused on SBAR contents, its importance, and its appropriate utilization.

Statistical analysis

Data regarding adherence to handoff according to the SBAR process were collected using an Excel sheet; data were then entered and analyzed using IBM SPSS Statistics software (version 26) (IBM Corp., Armonk, NY). Each item of the SBAR sheet was coded as either “documented” or “not documented.” The frequency of documentation of each item was presented as a percentage of the total number of handover occurrences, and the overall percentage of adherence to each of the four components of SBAR was recorded. Descriptive statistics were instituted to explore participants' perceptions and satisfaction with the SBAR form implementation. Participants' responses were graded in a three-point Likert scale (agree, neutral, and disagree), and expressed as frequencies and percentages.

## Results

In the first cycle, the study analyzed the handoff practices in 19 occurrences of 11 patients admitted with critical illnesses. While the second cycle assessment analyzed handoff forms of 17 different patients, constituting a total of 33 handoff occasions. Clinical cases that were included in the study are summarized in Table 1.

**Table 1:** Clinical cases included in the study.

Clinical cases in the first cycle	Clinical cases in the second cycle
Severe malaria	Severe malaria
Febrile convulsions	Sepsis
Active bleeding	Acute severe asthma
Congestive heart failure	Diabetic ketoacidosis
Sickle cell anemia	Aspiration pneumonia
Subarachnoid hemorrhage	Pure red cell aplasia
Post-infectious glomerulonephritis	Anemic heart failure
	Febrile convulsions
	Down syndrome complicated by heart failure
	Acute lymphocytic leukemia

Regarding adherence to handoff protocol using the SBAR form in the first cycle, out of the 10 items in the "Situation" component, only four were documented, with each item documented only three times from the total 19 handoff processes (Table 2). Furthermore, items in the "Background," "Assessment," and "Recommendation" components were never documented during any of the first cycle handoff processes (Tables 3-5).

**Table 2:** Item documentation rate regarding the "situation" component.

Situation	Item	Pre-intervention (n=19)	Post-intervention (n=33)
	Date	0 (0.0%)	31 (93.9%)
	Unit	3 (15.8%)	32 (97%)
	Doctor	0 (0.0%)	32 (97%)
	Phone	0 (0.0%)	25 (75.8%)
	Patient	3 (15.8%)	33 (100%)
	Age	3 (15.8%)	29 (87.9%)
	Weight	0 (0%)	27 (81.8%)
	Diagnosis	3 (15.8%)	32 (97%)
	Ward number	0 (0.0%)	22 (66.7%)
	Date of admission	0 (0.0%)	30 (90.9%)

**Table 3:** Item documentation rate regarding the "background" component.

Background	Item	Pre-intervention (n=19)	Post-intervention (n=33)
	Relevant medical history	0 (0.0%)	29 (87.9%)
	Relevant family history	0 (0.0%)	23 (69.7%)
	Recent investigations results	0 (0.0%)	26 (78.8%)
	Current medications/fluids	0 (0.0%)	30 (90.9%)
	Allergy	0 (0.0%)	30 (90.9%)

**Table 4:** Item documentation rate regarding the "assessment" component.

Assessment	Item	Pre-intervention (n=19)	Post-intervention (n=33)
	Pulse rate	0 (0.0%)	27 (81.8%)
	Respiratory rate	0 (0.0%)	27 (81.8%)
	Temperature	0 (0.0%)	26 (78.8%)
	Oxygen saturation	0 (0.0%)	16 (48.5%)
	Blood pressure	0 (0%)	4 (12.1%)
	Glasgow coma scale	0 (0.0%)	23 (69.7%)
	IV lines sites	0 (0.0%)	22 (66.7%)
	Current status of the patient	0 (0.0%)	30 (90.9%)

**Table 5:** Item documentation rate regarding the "recommendation" component.

Recommendation	Item	Pre-intervention (n=19)	Post-intervention (n=33)
	Need to change plan	0 (0.0%)	27 (81.8%)
	New plan	0 (0.0%)	14/22 (63.6%)
	Regular follow up	0 (0.0%)	23 (71.9%)
	Pending investigation	0 (0.0%)	20 (60.6%)

The post-intervention assessment (second cycle) revealed remarkable improvement in the documentation of patients' information during the handoff process (Tables 2-5). A significant improvement in adherence to all sections of the SBAR form was noted, with more pronounced adherence to the documentation of the “Situation” (88.8%) and “Background” (83.6%) sections, compared to the “Assessment” (66.3%) and “Recommendation” (69.5%) sections (Table 6).

**Table 6:** Overall documentation rate for each of the four components of the SBAR form. SBAR, Situation, Background, Assessment, and Recommendation

		First cycle	Second cycle	Standard
Component	Situation	12/190 (6%)	293/330 (88.8%)	90%
	Background	0/95 (0.0%)	138/165 (83.6%)	90%
	Assessment	0./152 (0.0%)	175/264( 66.3%)	90%
	Recommendation	0/76 (0.0%)	84/121 (69.5% )	90%

In addition, a survey assessing the perception of the SBAR tool was distributed to all department doctors practicing in the pediatric ward; the total of which was 48 doctors, comprising 28 house officers (58.3%), 10 medical officers (20.8%), and 10 registrars (20.8%), nearly two-thirds being females (60.4%). Respondents were divided in half regarding prior exposure to the SBAR tool, however, the majority (>85%) believed the SBAR form is useful in patients' management, safety, physicians' communication, and accountability. Furthermore, the majority (83,3%) felt comfortable with the use of the SBAR tool for handoff communication facing no difficulty understanding its contents; nonetheless, nearly one-quarter mentioned that filling the SBAR was time-consuming to some degree (Table 7).

**Table 7:** Perception of doctors regarding the SBAR form. SBAR, Situation, Background, Assessment, and Recommendation

	Disagree	Neutral	Agree
	N (%)	N (%)	N (%)
SBAR form application promotes effective communication among healthcare professionals	1 (2.1%)	5 (10.4%)	42 (87.5%)
SBAR form application helps in expressing ideas in a clear concise manner	1 (2.1%)	9 (18.7%)	38 (79.2%)
SBAR form application helps gain new insights and reflect on current patient management to improve care	0 (0%)	7 (14.6%)	41 (85.4%)
SBAR form application promotes accountability in patient care	1 (2.1%)	5 (10.4%)	42 (87.5%)
Information relevant to patient care is captured in SBAR form	2 (4.2%)	4 (8.3%)	42 (87.5%)
SBAR form will improve patient safety	1 (2.1%)	4 (8.3%)	43 (89.6%)
Comfortable with using SBAR form for handover communication	2 (4.2%)	6 (12.5%)	40 (83.3%)
Difficulty in understanding the content of the SBAR form	35 (72.9%)	7 (14.6%)	6 (12.5%)
It's time-consuming to fill SBAR form	25 (52.1%)	12 (25%)	11 (22.9%)

## Discussion

This audit aimed to improve handoff communication between doctors in the pediatric department by integrating the SBAR form into doctors’ practice. The practice of patient handoff in developing countries is thought to rely mainly on verbal communication with the scarce implementation of standardized, written patient handoff [9]. This is supported by the findings reported in our audit. On the contrary, in developed countries with advanced health systems, standardized patient handoffs are considered a vital practice, which led to the endorsement of the SBAR form in patient care that was linked to better health outcomes [10]. In addition, verbal handoff is more error-prone, while a standardized, written handoff ensures accuracy and promotes accountability [11]. In a previous recent study conducted by Blazin et al., decreased handoffs error rates were observed after the implementation of a standardized written handoff form [12].

This audit revealed poor adherence to written handoff practices among study participants, which consequently increased significantly after conducting regular training sessions. Items in the “Situation” component were poorly documented in pre-existing practices, however, a pronounced improvement was observed following the intervention. Other previous similar studies conducted by Scolari et al. [13] and Achrekar et al., among nursing professionals also reflected similar results with regard to the “Situation” components [9]. Regarding items in the "Background" component, there was also a noticeable improvement following the intervention, which was also noted in a study conducted by Achrekar et al. [9]. However, a lower percentage of adherence to this component was reported by Scolari et al. [13]. The "Assessment" component of the SBAR form displayed the lowest percentage of improvement in this study and a similar conclusion was reported by Scolari et al. [13]. Achrekar et al. reported a small difference in improvement within this component; however, this could be explained by the initial good practice observed in his study regarding this component [9]. The scarcity of resources, including tools like pulse oximeters, sphygmomanometers, and thermometers in the pediatric ward, could explain the lowest increase in adherence to the “Assessment” component. With regard to the "Recommendation" component, there was increased adherence to all its components in the second cycle, but this increase was significantly less in comparison to that reported in the “Situation” and “Background” sections. In the study conducted by Achrekar et al., no significant difference was reported in the “Recommendations” section [9].

Previous evidence has demonstrated the importance of SBAR form in patient safety and outcomes. Randmaa et al. reported that the critical incidence reporting system (CIRS) events due to communication breakdowns decreased from 31% to 11%. Also, Christie et al. found a decrease in hospital mortality before and after their study [14-16].

Healthcare professionals' view of the importance of the SBAR tool is highly crucial, as it directly affects their adherence to uitilizing this tool for proper patient handoff. Overall, doctors displayed a positive attitude regarding their perception of SBAR form implementation, a finding similar to previous literature reports [13, 17-18]. However, a notable proportion of doctors raised issues regarding time constraints in filling the SBAR form; these issues were also reported in previous studies conducted among nurses [13, 19], which warrants a review of the simplicity of the SBAR form structure, additionally as a number of doctors also revealed difficulty understanding the content of the SBAR form. Most of the doctors in this survey were comfortable with adding the SBAR form as a tool for handoff communication, while Nagammal et al. reported that only 1% of the nurses had poor satisfaction regarding using the SBAR form [17]. Furthermore, previous literature indicates that using communication tools like SBAR does not consume time or effort to complete, helps in the efficient delivery of patients’ information, encourages healthcare professionals’ collaboration, and decreases the rate of miscommunication errors [13, 19-23].

 Strengths and limitations

This study has several strengths. Firstly, most of the previous studies focused on simulation and e-learning training methods to assess the quality of SBAR form as a tool for communication [24-27]. However, this study assessed the quality of SBAR utilization in a real-life setting. Secondly, the study assessed the SBAR form components generally and then each component separately, furthermore, it evaluated the perception of doctors regarding SBAR form, all of this aids in detecting the areas of weaknesses in most need of improvement.

On the other hand, the study had some limitations. The SBAR form is a self-report tool, and some doctors might have had difficulty understanding the contents required for documentation, therefore, the accuracy of the entry of SBAR data was questionable. This study focused solely on critically ill patients, so the sample size was small and hence cannot be generalized. Most notably, patient care outcomes in terms of the average length of stay and other clinical outcomes related to communication were not evaluated. Another limitation of this study was the lack of prior similar research in Sudan or among doctors which made it difficult to compare the obtained data and draw conclusions.

## Conclusions

In general, the results of the second cycle showed that the introduction of training sessions had a positive impact on the use of SBAR forms and adherence to filling them, which helped doctors capture all relevant information about patient care. Furthermore, doctors displayed a positive response regarding the use of the SBAR form, nevertheless, further studies are needed to assess their compliance with the handoff tool use.

## Appendices

                                                                           Ribat Teaching Hospital

                                                                            Pediatrics Department

                                                              SBAR sheet: Handoff communication 

Situation:

 *Date:........………………..                                                              Unit:,..........……………………………………

*Doctor:............... ……………                                                          Phone:..............……….. 

*Patient Name :...................… .………    Age:...........…. . .      Weight………………………………………………..

*Diagnosis:……………………………………………………………………………………………………………………………………

….……………………………………………………………………………………………………………………………………………

*Ward number:………………DOA:……………………………………………………………………………………………………..

Background:

*Relevant Medical History :...........................................................………………………………………………………

….……………………………………………………………………………………………………………………………………………..

*Relevant Family History :...........................................................……………………………………………………..

….…………………………………………………………………………………………………………………………………………………….

*Results of Recent investigations :………………………………………………………………………………………………….

....…….….……………………………………………………………………………………………………………………………………….

….…………………………………………………………………………………………………………………………………………………..

*Current Medications or fluid :................................................................…………………………………………

.........,......................................................................................................…………………………………………….

….……………………………………………………………………………………………………………………………………………………….

*Allergy:Yes………No……IF yes specify………………………………………………………………………………………………..

*Others :……………………………………………………………………………………………………………………………………………..

Assessment:

*Current Vital signs :-

-PR=........                                        - O2 sat=.......       -GCS (if needed)=......… BP:………………..

-RR=........                                         - TEMP=.....…       .IV Line sites :.……………………………………………

*Current status of the patient is:1/ Stable(   ) 2/Deteriorating(    ) specify…………………………………………….

…………………………………………………………………………………………………………………………………………………………….

Or 3/ there is any clinical  problem may occur(    )specify…………………………………………………………………..

….………………………………………………………………………………………………………………………………………………….………

Recommendations:

*Need to change ,add or modify management plan?yes(. ) No(. ) if yes ,based

on:…………………………………………………………………………………………………………………………………………….

….…………………………………………………………………………………………………………………………………………….

*The added drug or modified plan: .................................................................................…………………….

..............................................................................................................................……………………………

..............................................................................................................................……………………………

….…………………………………………………………………………………………………………………………………………………..

*Clinical monitoring for:.............................................................................................…………………..

*Any pending investigation:.............................................................................................………………....

................................................................................................................................……………………………..

*Other:….…………………………………………………………………………………………………………………………………………:
